# Supplementary material for: Modelling thalamocortical circuitry shows that visually induced LTP changes laminar connectivity in human visual cortex
Source: PLoS Comput Biol. 2021 Jan 21;17(1):e1008414. doi: 10.1371/journal.pcbi.1008414 (PMC7853500; doi:10.1371/journal.pcbi.1008414)
Supplement: S1 Fig — (DOCX) [file pcbi.1008414.s001.docx]

**Supplementary Material: S1 Fig**

***Modelling thalamocortical circuitry shows visually induced LTP changes laminar connectivity in human visual cortex***

*Event Related Potential* *Maximum Intensity Projection*


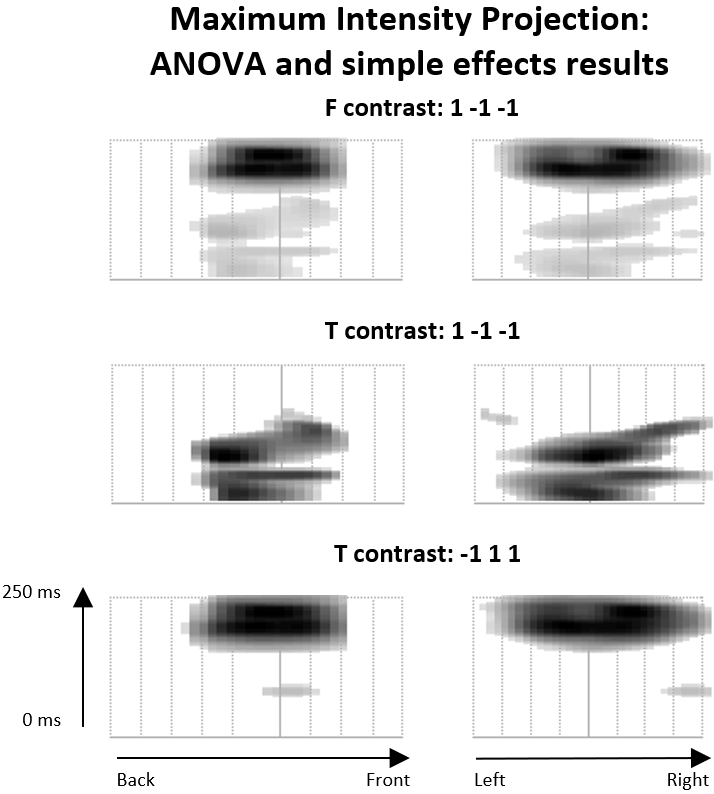


Maximum intensity projection (MIP) image from SPM12 family-wise error corrected results shows spatial and temporal distribution of the significant clusters. Note: Standard glass brain MIP not shown as occipital-parietal selection of electrodes is not rendered accurately on the glass brain.
