# Supplementary material for: Modelling thalamocortical circuitry shows that visually induced LTP changes laminar connectivity in human visual cortex
Source: PLoS Comput Biol. 2021 Jan 21;17(1):e1008414. doi: 10.1371/journal.pcbi.1008414 (PMC7853500; doi:10.1371/journal.pcbi.1008414)
Supplement: S2 Fig — (DOCX) [file pcbi.1008414.s002.docx]

**Supplementary Material: S2 Fig**

***Modelling thalamocortical circuitry shows visually induced LTP changes laminar connectivity in human visual cortex***


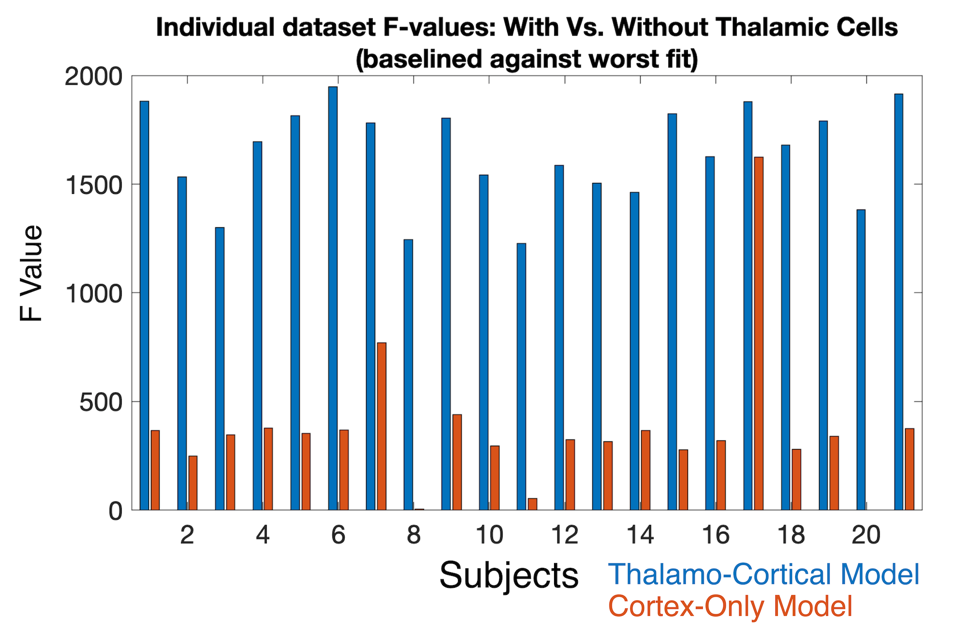


*FFX and RFX comparing the thalamo-cortical and cortical-only models for explaining the combination linear-nonlinear model of the tetanised data. The thalamo-cortical model is clearly superior in all datasets.*
