# Supplementary material for: Modelling thalamocortical circuitry shows that visually induced LTP changes laminar connectivity in human visual cortex
Source: PLoS Comput Biol. 2021 Jan 21;17(1):e1008414. doi: 10.1371/journal.pcbi.1008414 (PMC7853500; doi:10.1371/journal.pcbi.1008414)
Supplement: S3 Fig — (DOCX) [file pcbi.1008414.s003.docx]

**Supplementary Material: S3 Fig**

***Modelling thalamocortical circuitry shows visually induced LTP changes laminar connectivity in human visual cortex***


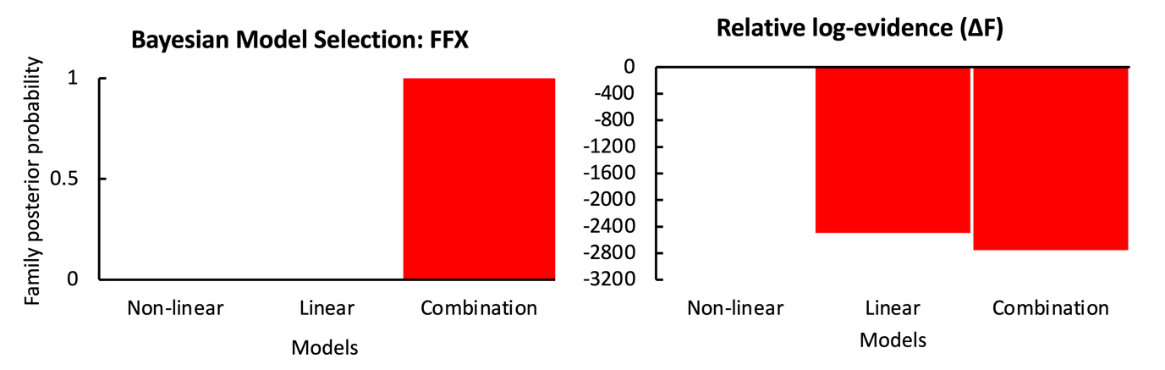


*FFX and RFX BMS for the non-tetanised data, demonstrating preference for the combinatorial linear-nonlinear model in explaining the non-tetanised data.*
