# Supplementary material for: Modelling thalamocortical circuitry shows that visually induced LTP changes laminar connectivity in human visual cortex
Source: PLoS Comput Biol. 2021 Jan 21;17(1):e1008414. doi: 10.1371/journal.pcbi.1008414 (PMC7853500; doi:10.1371/journal.pcbi.1008414)
Supplement: S1 Text — (DOCX) [file pcbi.1008414.s004.docx]

**Supplementary Material: S1 Supplementary Methods**

***Modelling thalamocortical circuitry shows visually induced LTP changes laminar connectivity in human visual cortex***

*Modelling*

*Parameterisation of the neural and observation models:*

Here we aim to set out how the model equations were parameterised in order to invert the model to the empirical ERPs. Elements of the equations which are parameterised are highlighted in **bold**. Starting with the conductance equation:

$$\varsigma_{n}=\gamma_{i,j}\sigma(\mu_{v}^{j}-V_{R}, \sum j)$$

Eq a.

Gamma is a (**parameterised**) sparse matrix of connectivity parameters between subpopulations, such that $\gamma_{i,j}$, describes the synaptic strength of the connection from population j to population i.

Lower case sigma, $\sigma$, represents the expected (average) firing rate of the source population j (sigma is a vector of 8 **parameters**, each corresponding to the firing rate of one of the populations). V_R_ is a fixed threshold of -40 mV for firing (not a parameter).

The second component of the conductance equation:

$$\dot{g}_{n}= \kappa_{n}\left( \varsigma_{n}- g_{n} \right)$$

Eq b.

This update scheme describes the evolution of the conductance, determined by the rate constant of channel n ($\kappa_{n})$ multiplied by the change in conductance (the output of equation a) minus the same quantity from the last time step. The rate constant (K) is a **parameterised** vector of length 4 (1 value each for AMPA, NMDA, GABA_A_ and GABA_B_ channels). These receptor ‘rates’ are common across all subpopulations.

The computed conductances for each channel (g_n_) permit calculation of the voltage equation (again, an update-scheme):

$$\frac{\mathrm{dV}}{\mathrm{dt}}=(\sum g_{n}\left( V-V_{n} \right)+u)/C$$

Eq c.

Here, V is the membrane voltage of the population. V_n_ is the reversal potential of channel n (fixed, not a parameter) and u is any endogenous or exogenous input current (a single **parameter**, in this experiment the mean of a Gaussian bump function in time). C is the membrane capacitance (**parameter** vector of length 8; one capacitance for each subpopulation).

Numerical integration of equation c leads to a membrane-potential (voltage) time series for each population. In DCM, this time-series is referred to as a hidden state, since it is not directly observed in the empirical data, rather an observation (aka forward) model is applied to this timeseries in order to compare it to the empirical EEG data.

*Observation model*

ERPs had already been extracted from visual cortex, hence this model has no spatial component to the observation model. Instead, the observation model, linking the population membrane potential timeseries to the EEG ERP, was a simple weighting of the contribution of each population (J) and a single electrode gain parameter (L).

J was fixed such that superficial pyramidal cells contribute the most to the signal (80% of their value) and L4 stellates, L5 pyramidal and L6 pyramidal contribute 20% of their absolute value. Interneurons and thalamic cells did not contribute directly to the observed signal, only through there interactions (and effects on) the other populations. As such J is not a ‘parameter’ since it was fixed during inversion. L (electrode gain) was a single value parameter.
