# Supplementary material for: Modelling thalamocortical circuitry shows that visually induced LTP changes laminar connectivity in human visual cortex
Source: PLoS Comput Biol. 2021 Jan 21;17(1):e1008414. doi: 10.1371/journal.pcbi.1008414 (PMC7853500; doi:10.1371/journal.pcbi.1008414)
Supplement: Attachment — Submitted filename: Sumner_et_al_Response_Revision1.docx [file pcbi.1008414.s005.docx]

**Dear Reviewers,**

**Thank you for reviewing our manuscript and for the constructive feedback and suggestions. We hope you will be pleased to see we have incorporated many of your suggestions into the paper. We believe the paper is stronger because of these additions and changes. The reviewer comments have been copied as written, with each response in bold below each point.**

**Yours sincerely,**

**On behalf of all authors,**

**Dr Rachael Sumner**

**Reviewer 1**

This paper describes the use of a thalamo-cortical DCM to determine the locii of LTP in a visual stimulation EEG paradigm. I greatly enjoyed reading this paper - I found the idea really interesting, and the design and analysis well executed. My main concern is that the model is not sufficiently probed using control analyses. However, if this concern was addressed, I would have no problem recommmending this paper for publication.

**Major comments**

More details should be provided about the source analysis. How many spatial, temporal modes were used? What was the sensor level covariance matrix? What forward model was used? I'm guessing that template surfaces and/or MRI volumes were used for the forward model. How were the fiducial coordinates defined?

**Our apologies, the lack of detail was an oversight. The following has been added:**

**“As individual MRI structural scans were not available, the template head model was used (based on the MNI brain) with the ‘normal’ cortical mesh. For the forward computation the standard head model option for EEG is the Boundary Element Method (BEM). Electrode locations used a standard template for the 64 channel ActiCap, including standardised fiducials. The source analysis was run on the time-window 100-220 ms to balance encompassing the major effects of the photic tetanus on the evoked response determined from the ERP analysis, with as narrow as possible time-window to limit the number of multiple comparisons. A 3-way repeated measures ANOVA was run (pre tetanus x early post-tetanus x late post-tetanus). A peak was selected that using the FWE-c F contrast [1 -1 -1] encompassed both early and late effects of the photic tetanus allowing a single node to be modelled. The selected peak was extracted from each participant as the local field potential (LFP), with a 5 mm spherical radius around the MNI coordinate using spm_eeg_inv_extract. Projecting the data into the brain as a local field potential (LFP) allows for the solving of the spatial forward model prior to solving the observation model, this is computationally more expedient for example by removing tissue conduction as a potential source of variation at the observation model fitting stage.”**

The main piece that I think is missing is any sort of control condition and model. Most crucially, what about post-tetanus VEPs to the grating orientation not used for the tetanus? Would the model correctly identify no changes in its parameters? I think it's fine if you don't want to include this in the ERP analysis (making it a 4-way ANOVA). I think you could do 2, 3-way ANOVAs, but I would mainly like to see what happens to the model when fit to this data.

**Thank you for this suggestion, during the drafting of this paper it did cross our mind too that this would be an interesting investigation. We decided to keep the focus on the modelling in this paper, so while we did not extend the ERP analysis (as this was meant to be just a quick confirmation analysis), we did run the model on the non-tetanised data. We hope you will be as interested as we were by the results:**

**“The equivalent analysis run on the non-tetanised data produced individual data fits that explained >90% variance for all participants for all models. An identical RM-MANOVA was run on the linear and non-linear model separately. The RM-MANOVA for the non-linear model revealed a significant of condition (*F*_(26,54)_=2.898, *p*=0.0001) using Wilk’s lambda λ=0.167. The univariate ANOVA result revealed a significant increase in ss🡪ss (*F*_(2,54)_=5.878, *p*=0.0197 FDR), ss🡪si (*F*_(2,54)_=6.772, *p*=0.013 FDR), and AMPA (*F*_(2,54)_=7.537, *p*=0.013 FDR). There was a significant decrease in sp🡪sp (*F*_(2,54)_=7.547, *p*=0.013 FDR).**

**The RM-MANOVA for the linear model revealed a significant effect of condition (*F*_(26,54)_=3.957, *p*=0.000012) using Wilk’s lambda λ=0.113. The univariate ANOVA result revealed a significant increase in si🡪ss (*F*_(2,54)_=13.026, *p*=0.00007 FDR), and rl🡪ss (*F*_(2,54)_=7.247, *p*=0.014 FDR).”**

**Discussion:**

**“Modelling was also carried out on the non-tetanised data and demonstrated evidence that the thalamo-cortical model is sensitive to the orientation of the tetanising stimulus. In the visual LTP ERP literature, the N1 has demonstrated input specificity to the spatial resolution and orientation of the tetanised grating (McNair et al., 2006; Ross et al., 2008; Sumner et al., 2020a). Specificity of LTP to the spatial resolution of sine gratings has also been shown in rodents (Cooke and Bear, 2010). The P2 does not demonstrate input specificity to these stimulus properties and it has been posited P2 may be sensitive to some other undetermined stimulus characteristic, as P2 is generated later in the visual processing pathway than N1 (Sumner et al., 2020b). Therefore, in the current study modelling the non-tetanised data was expected to cause broadly similar changes to the tetanised data, however, representing the contribution of orientation specific LTP to the evoked response data, less parameters would be expected to be significant. This is exactly what was found. Half as many linear parameters were significantly increased (si to ss, and rl to ss). Half as many of the same parameters as in the tetanised non-linear model were significantly increased for the non-tetanised non-linear model (ss to si, and AMPA). Notably absent from the non-tetanised stimulus compared to the tetanised stimulus was significant modulation of the connection from layer IV to III via ss to sp. Additionally, the sp to sp connection moved from a significant linear increase in the tetanised only model, to a non-linear decrease. This is speculated to represent concurrent transient LTD in superficial layers in the non-tetanised data. The additional self-gain parameter ss to ss was significantly increased for the non-linear model.**

**Overall, these data from the non-tetanised model output provide evidence that the presented thalamo-cortical model is sensitive to the orientation of the tetanising input grating. In particular the increase in rl to ss, and also AMPA parameters indicate that LTP still occurs in the non-tetanised data, although to a lesser extent (in fewer populations of cells). Evidence of connectivity and AMPA changes consistent with LTP in the non-tetanised model is interpreted as the contribution of cortical microcircuitry involved in later visual processing. This interpretation could be tested in future research using hemispheric input specificity via ocular occlusion, whereby no significant LTP driven parameter modulation would be expected to occur in visual cortex of the non-tetanised hemisphere (based on ocular dominance and LTP reviewed by Cooke and Bear (2014) and visual LTP results from Teyler et al. (2005)).”**

**Additional figure in the discussion:**

***Figure 7. Difference in significant parameter changes for the tetanised compared to non-tetanised model. Note. Tetanised model results are identical to depiction in Figure 6 and are provided here for side-by-side comparison. See* *Figure 2 for key of parameters.***

What is required for the model to capture the post-tetanus VEP changes? What if, for example, you held constant the ss->sp, ss->si, and sp->dp conducatnces? Would the model still be able to fit the data by allowing other parameters to vary? If so, does this mean that the choice of which variables to fix has a strong impact on the results?

**Yes, it is likely if we had only allowed the parameters to vary that had specific hypotheses the model would still have fit. Other models such as the cmm_NMDA, and CMC models could be termed sufficiently “biologically plausible” to recapitulate LTP driven effects. However, we would expect that ours would be superior as it is more accurate.**

**We had designed the study in a way that addresses the reviewer’s concern about the impact of specifically choosing certain parameters on the results, but realise we did not describe this as well as we should have. The model recapitulates an entire system so we chose a wider range of parameters than we expected would describe LTP (by basing it on basic information transfer in the visual system) meaning the model could have fit to any number of parameters. The parameters may not have included the parameters we expected. Further, via the contrasts, connectivity could have decreased and not increased (and did for one parameter).**

**We now describe this better in the introduction:**

**“**The model was employed in the current study with the aim of capturing the induction of LTP in visual cortex following ~9Hz visual stimulation in the classical visual LTP paradigm (Sumner, Spriggs, et al., 2020). **It was hypothesised that the changes in laminar connections would be in the same locations as LTP driven changes found by Heynen and Bear (2001) and Huang et al. (2014). This includes an increase in excitatory connectivity projecting from thalamus into layer IV, and from layer IV into layer III. However, we did not restrict the model to only use these parameters to describe the data. We attempted to balance computational demands with complexity, and allowed all available parameters to vary in layer II/III and IV, as well as all thalamic projections to these layers, reminiscent of the Douglas and Martin (1991) model of basic visual processing.** This meant while less well documented the model will allow for the inhibitory changes to occur, potentially as a result LTP occurring on inhibitory interneurons. LTP on GABAergic interneurons has been found in hippocampus and cortex (Kullmann & Lamsa, 2011), including following TBS to visual cortex (Sarihi et al., 2008). LTP on GABAergic interneurons related to visually induced LTP may or may not be NMDA dependent and such findings will be exploratory.

**The specifying of a wide range of parameters additionally allows for the model to recapitulate changes that may be inconsistent with LTP or not include any of the hypothesised connectivity changes, for example, decreases in connectivity. The study is therefore a hypothesis driven exploration of visually induced LTP, that could plausibly provide alternative explanations for the modulation of ERPs seen in the visual LTP paradigm.”**

Are there significant differences in any of the parameters when comparing the early and late conditions (in addition to comparing each to the pre-tetanus condition)?

**We chose to look at the univariate ANOVAs as post-hoc testing to minimise the number of multiple comparisons to correct. It also provides a test of the effect of condition (pre-tetanus, early post-tetanus, late post-tetanus) for each of the contrasts (non-linear, linear). Furthermore, the contrast that differentiates the linear and non-linear models forces a relative difference between conditions when the beta-weights from the model parameter estimate are converted to units siemens. This does not cause a difference that is not in the beta-weights to emerge, but it does mean that if the univariate ANOVA is significant, then the early vs late conditions are different from each other, as well as the pre vs early and pre vs late.**

**Minor comments**

Make sure layer/cell acronyms are defined

"Thus, providing the basis for the development of biologically plausible computational models of the canonical microcircuit that estimate the neural causes of EEG signal."

This sentence has no subject

**This is now :**

**“The empirical evidence for basic principles governing connectivity in the brain provides the basis for the development of biologically plausible computational models of the canonical microcircuit that estimate the neural causes of EEG signal.”**

"potentially as a result LTP occurring"

should be

"potentially as a result of LTP occurring"

**This has been fixed**

"Where potentiation 30 minutes post-tetanus onward is likely to predominantly reflect E-LTP, and may contain some products of the conversion to L-LTP (Sumner et al., 2020; Sumner et al., In Press)."

Sentence fragment

**This is now:**

**“In contrast, potentiation observed 30 minutes post-tetanus onward is likely to predominantly reflect E-LTP, and may contain some products of the conversion to L-LTP (Sumner, McMillan, et al., 2020; Sumner, Spriggs, et al., 2020).”**

"and a single pool on inhibitory"

should be

"and a single pool of inhibitory"

**This has been fixed.**

**Reviewer 2**

The authors present a validation of a thalamocortical model of the microcircuit structure of visual cortex, under the assumption that the observed changes in visual evoked potential are directly correlated with the columnar organization and functionality changes of specific excitatory and inhibitory neurons, in basal photic stimulation and post photic-tetanus conditions.

This study is interesting since photic induced VEP-LTP, can be used as landmark on neurological disorders and pharmacological treatment, as previously shown by the same authors (Summer, 2020). But the question remains about if those changes are also reflected at the neuronal network level and this paper provided some evidence in that direction.

However, there are some issues to be clarified before the publication.

**Major**

The authors enthusiastically state that the evidence from previous work (Heynen and Bear, 2001(rat); Huang, 2014(monkey), which does not use photo stimulation) confirms the hypothesis proposed by them. At best this statement represents only a coincidence of cortical sinks and should not be taken as a validation of the authors' experimental results under any circumstances. A clearer discussion of this point is required.

**We have added further clarification that the paper by Huang et al. (2014) used TBS and not photic tetanus (where Heynen and Bear (2001) used TBS and photic).**

**The purpose of drawing comparisons between the animal literature and our findings was to determine that the modulations of model parameters were occurring in key sites that have demonstrated evidence of LTP driven changes in rodents and in primates already. Indeed Heynen and Bear (2001) describe the cortical sinks as evidence of increases in excitatory connectivity between these layers.**

**We accept that on reflection we may have over-generalised between our findings and the papers in question in the discussion, especially by using words like “replicating” and “validation”. We have toned this down throughout and added to the discussion that:**

**“Future steps to validate the model may include adapting it and applying it to invasive animal research on LTP in the visual system. This could be done using data such as that already acquired in Heynen and Bear (2001) and Clapp et al., (2006) similarly to the use of DCM in rodent LFP recordings in Moran et al. (2015)”**

**The best validation would be to have a combination of surface recordings and laminar specific recordings. Even better with drugs that block LTP.**

A proper supporting paper are required to show the relevance of high-frequency visual stimulation to induce long lasting effect at cortical LTP, a unpublish paper should not be cited as supporting evidence across several section of the paper: “Sumner, Spriggs, Muthukumaraswamy, & Kirk, In Press”.

**Our apologies, we were expecting the paper would be available online as it had been accepted for publication prior to submission and we had thought it was a matter days away from being online at the time. However, we experienced unexpected delays and issues at the proofing stage. If we had known this was going to happen we would have at least provided a preprint of the paper and will be more cautious in future.**

**Minor**

Please check the references section, some have the DOI annotated as URL and others not. Also there is missing information in some of them, for example: “Adams, N. E., Hughes, L. E., Phillips, H. N., Shaw, A. D., Murley, A. G., Nesbitt, D., . . . Rowe, J. B. (2020). GABAergic dynamics in human frontotemporal networks confirmed by pharmacomagnetoencephalography. Journal of Neuroscience, (XXXXX)?”

**Thank you for pointing this out. We have updated the references to be consistent.**

**Reviewer 3**

This manuscript is entitled: “Modelling thalamocortical circuitry shows visually induced LTP changes laminar connectivity in human visual cortex”. It builds on recent work for the development of biologically plausible generative models of EEG data, enabling to relate experimental modulations of scalp recordings to changes in laminar connectivity parameters at the microcircuit level. These Dynamic Causal Models (DCMs) have been extensively developed and refined over the last 10 years or so and represent an important paradigm shift to test mechanistic hypothesis, in health and disease, and evaluate treatments, non-invasively.

Precisely, this paper intends to contribute to this endeavour by bridging the gap between invasive studies in animal models and human in vivo essays. Therefore, it focuses on visually induced LTP changes using a previously published EEG tasks in adult volunteers. Part of the data presented here (female subjects) have been published previously.

Both for its objectives and for its original methodology, this paper is of importance to the field.

However, to fully validate the approach and the obtained findings, a crucial step is to demonstrate the identifiability of the proposed model as well as to convincingly show that the identified model is indeed plausible. In that respect, I have several questions and concerns. I have no doubt the authors can answer them which I believe will make the paper quite stronger and of interest to a broader readership.

**Major**

The authors have extended the microcircuit model previously published to cover their needs, given knowledge from animal studies. This makes perfect sense. However, those complex dynamic models have many parameters and especially when extended like here, they call for a careful validation. In particular:

- EEG is known to have a poor spatial resolution (namely w.r.t. MEG). It might well be that the well-motivated temporal constraints entailed by the model structure and interactions are sufficient to provide a plausible explanation for the observed experimental modulations of scalp EEG data. However, it might also be that a very large repertoire of alternative plausible and very different (possibly simpler) models would fit the data just as well. I am not suggesting here that the authors should consider an infinite model space. Some important factors/dimensions should be considered and investigated: for instance, is the thalamus compartment needed? (having in mind that activity in the thalamus is not directly ‘seen’ by EEG).

**It is the nature of modelling that other models could plausibly fit the data and produce interesting results, including far simpler models. However the objective of this thalamo-cortical model is to parameterise thalamic input into the system, not by implying that the thalamus has direct signal, but by establishing how changes to the thalamic parameters could affect the observed data. It may be that future studies on LTP choose to use simpler models for various reasons, computational expediency for example. However, there is evidence to suggest that the presented model is superior to close, simpler, alternatives using Bayesian Model Selection (BMS).**

**In Shaw et al. (2020), the thalamo-cortical model is compared to the standard canonical microcircuit model offered “out of the box” with the DCM framework. The comparison with BMS determines with very strong evidence that the thalamo-cortical model is a better model.**

**In the current study we now run BMS on the thalamo-cortical model, with the thalamus and then without,** **replacing thalamic input with a gaussian bump function into layer 4, as is standard in DCM.**

**In the methods:**

**“Next, due to the relative novelty of parameterising and modelling thalamic connectivity, the additional step of using BMS to compare the winning model a model with no thalamic compartment is provided, replacing thalamic input with a gaussian bump function into layer 4.** **BMS has already determined that thalamo-cortical model is superior to simpler alternatives such as the 4 population CMC model this model was built on (cmm_NMDA) (Shaw et al., 2020a).”**

**And results:**

**“For the winning, combination linear-nonlinear model, we explored the possibility that a cortex-only version of the model (i.e. without the thalamic populations) would suffice. Using BMS, we compared the thalamic model to a cortex only model using BMS, which clearly showed very strong evidence for the model with thalamic cells (FFX=1; RFX-BOR = 0.1e-4; pxp with thalamus = 0.9565, without = 0.0435). Supplementary Figure 1 provides the relative log-evidence for each model.”**

- Similarly, Bayesian Model Selection could further be used to formally test about the ability to estimate certain parameters, e.g. those who have been found to vary but whose modulations were not predicted by previous findings in animal models.

**One of the challenges is also that even if BMS determined the model is better with the unanticipated parameter changes than without, it is still not clear whether the modulations are LTP driven or some other experimental effect on connectivity. The most ideal validation step would be to utilise the model on animal recording data that demonstrates some other additional empirical evidence of LTP in the data (such as a condition that uses an LTP blocking drug) and we provide some discussion of this:**

**“Future steps to validate the model may include adapting it and applying it to invasive animal research on LTP in the visual system. This could be done using data such as that already acquired in Heynen and Bear (2001) and Clapp et al. (2006), similarly to the use of DCM in rodent LFP recordings in Moran et al. (2015).”**

- In that respect, a few simulations that would show the difference in the repertoire of dynamics that the alternative models can cover would be highly informative (e.g. a model with an explicit vs. implicit thalamus compartment; or a model with vs without a non-linear effect). In line with recommendations in terms of model falsifiability [1].

**We agree this would be informative, and we believe we have addressed this as part of the reviewer's first point by running the BMS on the model with and without the thalamus compartment to the model. The addition of the thalamic connectivity is the most novel part of the model. As also indicated in an above point we do refer to previous studies that validated the use of non-linear NMDA receptor parameterisation, and now also refer to the explicit comparison of the current model with the more basic 4 population cmm_nmda model.**

- A related issue is the choice that the authors made for the data to be fitted by the DCMs. Instead of directly fitting the scalp data as commonly done with DCM for ERPs (that is fitting all sensors or their main spatial and temporal modes), the authors proceeded in two steps by first source-reconstructing the activity in a specific ROI of the visual cortex and fitted this reconstructed activity. Why ? I wonder if this may come with a critical loss of information ?

**Part of the decision to do this is to cut down on computational demands by reducing the dimensions of the inverted model. Instead of inverting a spatial-temporal model, projecting the data into the brain as a local field potential makes it a temporal model only. The spatial model is solved first. This is not an uncommon approach for DCM of canonical microcircuit models (Shaw et al., 2020b; Sumner et al., 2018; Muthukumaraswamy et al., 2015; Gilbert and Moran, 2016; Bhatt et al., 2016).**

**It is the case that this leads to some loss of information and we discuss this more in the following point.**

- Actually, the curves on Figure 4 tends to support this claim, as one can notice that the reconstructed dynamics in the chosen source do not exhibit all the experimental effects reported at the scalp level. In particular, there seems to be no effect at the N1 latency (which b.t.w seems to be shifted/postponed compared to the one at the scalp level).

**There was admittedly some loss of experimental information, however, this was expected when choosing a single node to represent ERP changes from the scalp that are known to incorporate a network of regions. On balance choosing a single node allowed for a much more simplistic model architecture but admittedly not without cost.**

**The shift in latency is not surprising, while the peak that survives multiple comparisons is at 132 ms - there is a more right lateralised cluster out to 170 ms uncorrected (P = 0.10 FWE-c) - the source and modelled results may in fact be doing a better job at showing this than POz.**

**Further to this, while we always present candidate ERPs from the electrodes in our papers, to demonstrate what we are measuring can be seen simply at the scalp - this is an example of SPM complicating things a little - SPM produces results at the field peak (using random field level); the results on the electrodes are not always a perfect representation.**

**The caption for Figure 5 (was 4) has also been edited to include explicit mention of the limitation and slight loss of information at the source level.**

**“**Figure 5. A) The VEP as recorded at electrode POz shows evidence of the differences pre-tetanus compared to both early post-tetanus and late post-tetanus found in the peak field effects. This includes **(least clearly shown)** a peak at 132 ms that was most significant in the early post-tetanus block, and **(most clearly shown)** at around 196 ms a peak that was most significant in the late post-tetanus block. B) Results of the source analysis looking at pre- vs early and late post-tetanus. Thresholded to p<0.05 uncorrected for illustration purposes. The crosshair lies over the literature selected peak coordinate [-36. -90, 4] in left middle occipital gyrus. C) The time related effects post-tetanus are recapitulated in both the source reconstructed evoked response, D) as well as the evoked response produced at the source by the model. **However, while expected, some loss of information from the scalp recorded ERP is apparent. The shift in latency of the early negativity is considered a more complete representation of the (usually more bilateral) N1 than shown at POz.”**

- Surprisingly, very few information is provided regarding the source reconstruction step. What kind of anatomical model was considered (individual or template anatomy) ? How did you define the ROI location for each individual? What temporal window did you consider to apply MSP? This is a critical issue (in relation to the previous comment), since MSP assumes stationarity over the whole inverted data time window. This contrasts with the aim here, with DCM, to capture the finesse of dynamics in the visual system.

**Our apologies, this was an oversight. Reviewer 1 also picked up on this and we have added the following detail (bold text added):**

“Sources analysis was carried out using Multiple Sparse Priors, as implemented in SPM12, for group inverse reconstruction (Litvak & Friston, 2008). **As individual MRI structural scans were not available, the template head model was used (based on the MNI brain) with the ‘normal’ cortical mesh. For the forward computation the standard head model option for EEG is the Boundary Element Method (BEM). Electrode locations used a standard template for the 64 channel ActiCap, including standardised fiducials. The source analysis was run on the time-window 100-220 ms to balance encompassing the major effects of the photic tetanus on the evoked response determined from the ERP analysis, with as narrow as possible time-window to limit the number of multiple comparisons. A 3-way repeated measures ANOVA was run (pre tetanus x early post-tetanus x late post-tetanus). A peak was selected that using the FWE-c F contrast [1 -1 -1] encompassed both early and late effects of the photic tetanus allowing a single node to be modelled. The selected peak was extracted from each participant as the local field potential, with a 5 mm spherical radius around the MNI coordinate using spm_eeg_inv_extract. Projecting the data into the brain as a local field potential allows for the solving of the spatial model prior to solving the observation model, this is computationally more expedient for example by removing tissue conduction as a potential source of variation at the observation model fitting stage.”**

**Additionally, (in bold) we have clarified in the result section that:**

“...a peak in left middle occipital gyrus (MOG) was selected from the literature (Spriggs et al., 2018) that was still within the significant area of activation for the current study: MNI [-36, -90, 4]. **This peak was extracted as a local field potential for all participants and described a mean variance of 77% (SD = 17%) of the scalp data (averaged across a cluster including and around POz comprising P1, Pz, P2, PO3, P4).”**

**It is an interesting point that MSP assumes stationarity, yet is commonly used for evoked potential data in the context of preparing it for DCM.**

Also, what data did you reconstruct exactly (all conditions together at once, the difference between two conditions…?). And did you make sure that the considered ROI corresponded, for each subject, to a source that was explaining enough of the variance of the scalp response?

**The first part of this comment is addressed in the above point. Including that** “... **a local field potential for all participants and described a mean variance of 77% (SD = 17%) of the scalp data (averaged across a cluster including and around POz comprising P1, Pz, P2, PO3, P4).”**

- Finally, you should report the individual Free Energies or Bayes Factors so that one can appreciate how your BMS results do reproduce over subjects and that the FFX approach is indeed justified.

**Reporting the Free energies wasn’t as clear/tidy as we would have liked, so we determined that the best approach was to run RFX in addition and present both results as in Shaw et al. (2019).**

**Addition to the methods:**

**“Random-effects (RFX) results are provided additionally to FFX as in (Shaw et al., 2019); RFX gives Bayesian Omnibus Risk (BOR) - the log-Bayes factor which provides the probability of a model’s frequency providing evidence for H1 over H0. A BOR of ≤ 0.25 is taken as strong evidence in favour of the winning model (Rigoux et al., 2014). Protected exceedance probabilities (pxp) provide a probability of the most frequent model, over all other tested models (Rigoux et al., 2014).”**

**And the results:**

**“FFX revealed that the combination model was the winning computational model (Figure 6). RFX BOR = 0.086 provides very strong evidence for the combination model (pxp = 0.49), closely followed by the linear model (pxp = 0.46), and ~16-fold weaker non-linear model (pxp = 0.03). While the result could be considered a split model win for the linear and combination model, the combination model does include the linear model, also extra correction is applied because the combination model contains extra degrees of freedom introduced by extra free parameters.”**

**This is then consistently used in the additional sections:**

***“Bayesian Model Selection on the thalamo-cortical model vs. a cortex-only model***

**For the winning, combination linear-nonlinear model, we explored the possibility that a cortex-only version of the model (i.e. without the thalamic populations) would suffice. Using BMS, we compared the thalamic model to a cortex only model using BMS, which clearly showed very strong evidence for the model with thalamic cells (FFX=1; RFX-BOR = 0.1e-4; pxp with thalamus = 0.9565, without = 0.0435). Supplementary Figure 1 provides the relative log-evidence for each model.**

***Bayesian Model Selection on the thalamo-cortical model for non-tetanised data***

**The equivalent analysis run on the non-tetanised data produced individual data fits that explained >90% variance for all participants for all models. BMS comparing the nonlinear, linear and combination models, for the non-tetanised data, also demonstrated preference for the combination model (FFX = 1, RFX pxp = 0.5063, see Supplementary Figure 2). As with the tetanised model there is evidence for split model win using RFX for the linear model pxp = 0.41, and non-linear was much weaker pxp = 0.09.”**

**Minor**

- How many trials per subject were rejected following the described pre-processing procedure ?

**The following has been added to the paper:**

**“This lead to the removal of on average 34.05 (SD=28.56) trials from the pre-tetanus condition, 19.50 (SD=10.75) from the early post-tetanus, and 39.85 (SD=16.10) from the late post-tetanus conditions, leaving and mean of 445.95, 220.50, and 200.15 trials per condition respectively.”**

- The authors used FCz as the reference. Usually an electrode that does not capture brain signals is preferred. Please comment and justify.

**As described here** [**https://pressrelease.brainproducts.com/referencing/**](https://pressrelease.brainproducts.com/referencing/)

**FCz is a standard online reference, pre-allocated to the cap type used in the current study. As explained in “ERP: Pre-processing” the data were re-referenced to the common average during pre-processing. The only downside to this is that FCz is not recording data, this is less of an issue for the visual LTP paradigm where the analysis focuses on occipital and parietal electrodes anyway.**

**To clarify this in text, the following has been added:**

**“As is standard for the ActiCap layout, FCz was used as an online reference and AFz as ground.”**

- In the methods section, ERP analysis paragraph, please specify explicitly the factors of the 3-way ANOVA.

**The following clarification has been added:**

**“A 3-way repeated measures ANOVA (pre tetanus x early post-tetanus x late post-tetanus)”**

- What do you mean by: “Simple effects analyses were carried out as appropriate” ?

**We have added clarification:**

**“Simple effects post-hoc analyses were carried out as appropriate to explore main effects and interactions.”**

- It would be helpful to show the spatio-temporal clusters that came out as significant in the ERP analysis.

**We have added the following figure to Supplementary Material, and direct readers to it in text:**

***“Supplementary Figure 3.* Maximum intensity projection (MIP) image from SPM12 family-wise error corrected results shows spatial and temporal distribution of the significant clusters. Note: Standard glass brain MIP not shown as occipital-parietal selection of electrodes is not rendered accurately on the glass brain.”**

**While these maximum intensity projection (MIP) images take a little to orient to spatially, because we constrain the electrodes analysed to P1, P2, P3, P4, P5, P6, P7, P8, Pz, PO3, PO4, PO7, PO8, PO9, PO10, POz, O1, O2, and Oz unfortunately SPM does not correct the MIPs and the clusters on the glass brain end up in the middle of the images. The glass brain would ordinarily more clearly demonstrate the spatial distribution of clusters. For example:**

**Is from the T-contrast -1 0 1 and ought to be located roughly where the red arrow indicates.**

- What software did you use for the RM-MANOVA ?

**The following has been added to the text:**

**“Statistics on the parameter estimates were carried out using SPSS 25 (Armonk, NY).”**

- A few typos:

• Page 11: “…, and a single pool OF inhibitory …”

• Better use Observation model everywhere instead of Observational model

• Page 19: “…the increase in variance causeD by…”

**Thank you for noticing these which had slipped by us. They have all been fixed and the manuscript has been inspected for any further typos.**

- I found some sentences a bit hard to read, including the title! Here are a few other examples:

**Thank you for bringing this to our attention, we have amended the sentences pointed out below and also re-read the manuscript and fixed any further problematic sentences we identified.**

**Regarding the title this was a fair point on reflection, we believe the addition of “that” improves the title:**

**“**Modelling thalamocortical circuitry shows **that** visually induced LTP changes laminar connectivity in human visual cortex**”**

“While less well documented the model will allow for the inhibitory changes to occur, potentially as a result LTP occurring on inhibitory interneurons.”

**Now:**

**“This meant while less well documented the model will allow for the inhibitory changes to occur, that may potentially be as a result of LTP occurring on inhibitory interneurons.”**

“In the first, pre-tetanus condition, both stimuli were presented in a random order 240 times at 1 Hz for 34.8 ms and takes approximately 8 minutes”

**Now:**

**“The first was a pre-tetanus condition; both stimuli were presented in a random order 240 times at 1 Hz for 34.8 ms. The pre-tetanus condition took approximately 8 minutes.”**

“The purpose of the ERP analysis was to confirm modulation of the ERP had occurred and so was conducted solely to demonstrate evidence of VEP modulation post-tetanus”

**Now:**

**“The purpose of the ERP analysis was to confirm and demonstrate evidence of VEP modulation post-tetanus.”**

“For this analysis we constrained the parameters that were allowed to vary (during model fitting) according to the Douglas and Martin (2004) canonical microcircuit, and included all of the additional parameters within layers II/III and IV, so this included … and meant the rest of the parameters were fixed”

**Now:**

**“For this analysis we constrained the parameters that were allowed to vary (during model fitting) according to the Douglas and Martin (2004) canonical microcircuit. The current model is more detailed than the Douglas and Martin (2004) canonical microcircuit so we also allowed all of the additional parameters within layers II/III and IV to vary. In summary:.... , were allowed to vary and the rest of the parameters were fixed”**

Reference

[1] S. Palminteri, V. Wyart, et E. Koechlin, « The Importance of Falsification in Computational Cognitive Modeling », Trends Cogn. Sci., vol. 21, no 6, p. 425‑433, juin 2017, doi: 10.1016/j.tics.2017.03.011.

**References**

Bhatt MB, Bowen S, Rossiter HE, et al. (2016) Computational modelling of movement-related beta-oscillatory dynamics in human motor cortex. *Neuroimage* 133: 224-232.

Cooke SF and Bear MF. (2010) Visual experience induces long-term potentiation in the primary visual cortex. *The Journal of Neuroscience* 30: 16304-16313.

Cooke SF and Bear MF. (2014) How the mechanisms of long-term synaptic potentiation and depression serve experience-dependent plasticity in primary visual cortex. *Philosophical Transactions of the Royal Society B: Biological Sciences* 369: 20130284.

Gilbert JR and Moran RJ. (2016) Inputs to prefrontal cortex support visual recognition in the aging brain. *Scientific Reports* 6.

McNair NA, Clapp WC, Hamm JP, et al. (2006) Spatial frequency-specific potentiation of human visual-evoked potentials. *NeuroReport* 17: 739-741.

Muthukumaraswamy SD, Shaw AD, Jackson LE, et al. (2015) Evidence that subanesthetic doses of ketamine cause sustained disruptions of NMDA and AMPA-mediated frontoparietal connectivity in humans. *The Journal of Neuroscience* 35: 11694-11706.

Ross RM, McNair NA, Fairhall SL, et al. (2008) Induction of orientation-specific LTP-like changes in human visual evoked potentials by rapid sensory stimulation. *Brain Res Bull* 76: 97-101.

Shaw AD, Hughes LE, Moran R, et al. (2019) In Vivo Assay of Cortical Microcircuitry in Frontotemporal Dementia: A Platform for Experimental Medicine Studies. *Cereb Cortex*.

Shaw AD, Muthukumaraswamy SD, Saxena N, et al. (2020a) Generative modelling of the thalamo-cortical circuit mechanisms underlying the neurophysiological effects of ketamine. *bioRxiv*: 688044.

Shaw AD, Muthukumaraswamy SD, Saxena N, et al. (2020b) Generative modelling of the thalamo-cortical circuit mechanisms underlying the neurophysiological effects of ketamine. *Neuroimage*: 117189.

Sumner RL, McMillan R, Spriggs MJ, et al. (2020a) Ketamine Enhances Visual Sensory Evoked Potential Long-term Potentiation in Patients With Major Depressive Disorder. *Biological Psychiatry: Cognitive Neuroscience and Neuroimaging* 5: 45-55.

Sumner RL, McMillan RL, Shaw AD, et al. (2018) Peak visual gamma frequency is modified across the healthy menstrual cycle. *Hum Brain Mapp* 39: 3187-3202.

Sumner RL, Spriggs MJ, Muthukumaraswamy SD, et al. (2020b) The role of Hebbian learning in human perception: a methodological and theoretical review of the human Visual Long-Term Potentiation paradigm. *Neurosci Biobehav Rev* 115: 220-237.

Teyler TJ, Hamm JP, Clapp WC, et al. (2005) Long-term potentiation of human visual evoked responses. *European Journal of Neuroscience* 21: 2045-2050.
